# Physician-staffed Ambulance Deployment: Comparative Response Time Analysis from a Slovak Pilot Project

**DOI:** 10.5811/westjem.52829

**Published:** 2026-05-19

**Authors:** Sedlak Marian, Petras Tomas, Berta Imrich, Ivanov Gaston, Karas Jozef

**Affiliations:** * Záchranná služba Košice, Košice, Slovak Republic; † Pavol Jozef Safarik University and Louis Pasteur University Hospital, Department of Trauma Surgery, *Košice*, Slovak Republic; ‡ Pavol Jozef Safarik University, Medical Education Centre, *Košice*, Slovak Republic; § Institute for Healthcare Analyses, Ministry of Health of the Slovak Republic, Bratislava, Slovak Republic; || Health Section, Ministry of Health of the Slovak Republic, Bratislava, Slovak Republic

## Abstract

**Introduction:**

Efficient allocation of physician-staffed emergency medical services (EMS) is crucial for optimal resource use in urban prehospital systems. The rendezvous model differs fundamentally from the traditional ambulance model: It deploys a lighter, nontransport-capable, passenger vehicle that may offer operational advantages, although comparative evidence with regard to traditional models remains limited. In this study we aimed to evaluate the impact of a physician-staffed rendezvous model on response times and physician-staffed crew availability within the EMS system in Košice, Slovak Republic.

**Methods:**

We conducted a retrospective, cross-sectional study, analyzing all primary responses by physician-staffed EMS units in the Košice region from January 2023–March 2025. In August 2024, one of three traditional, physician-staffed transport units was replaced by a physician-staffed rendezvous unit, yielding a post-intervention system with two transport units and one rendezvouz unit, a faster, physician-staffed non-transport vehicle that provides specialized medical care on scene. We extracted time intervals in minutes—response time, time on scene, time to transport initiation, and total time until crew availability—from the national EMS database and compared them to the pre- and post-introduction of the rendezvous model. We analyzed data using non-parametric statistical tests (Mann-Whitney U test, Kruskal-Wallis test), and we conducted a multivariable ordinary least squares regression to adjust for potential confounders.

**Results:**

Of 11,347 eligible cases, 11,094 met the inclusion criteria (8,389 patients treated during the pre-intervention period and 2,705 during the post-intervention period). Of these, 488 patients (4.4% of the overall cohort and 18.0% of the post-intervention cases) were managed by the rendezvous unit. Following rendezvous unit implementation, the mean response time was reduced compared to that of the standard physician-staffed transport units (−0.77 minutes per response; *P* < .001). The rendezvous unit demonstrated reductions in time to crew availability across districts, with absolute decreases ranging from 11.12 to 18.01 minutes; these differences were statistically significant in all districts except the undefined/border region (*P* < .001 for each comparison). The time spent on scene was slightly longer for the rendezvous unit in most districts, although these differences did not reach statistical significance. The time to transport initiation showed mixed trends. Ordinary least squares regression confirmed the independent association between rendezvous unit implementation and shorter response times.

**Conclusion:**

Replacing a standard physician-staffed ambulance with a lighter, faster, non-transport rendezvous vehicle improved operational efficiency by reducing response times and expediting physician-staffed crew availability. These findings suggest that the rendezvous model can enhance system-level performance in urban EMS settings by supporting more flexible physician deployment and informing decisions on resource allocation within tiered prehospital systems.

## INTRODUCTION

Emergency medical services (EMS) worldwide face increasing pressure to deliver timely care amid rising call volumes, constrained resources, and growing urban congestion. Optimizing the deployment of advanced prehospital resources, particularly physician-staffed units, has become a critical operational challenge for modern EMS systems. The EMS network in the Slovak Republic comprises 328 strategically located ambulance stations, functioning within a two-tiered care system—crews with physicians and crews with paramedics—covering both ground and air transport services.^1^

Physician-staffed responses represent a minority of total EMS activations and are primarily reserved for the most severe or diagnostically complex cases, where advanced decision-making and invasive interventions may be required. These crews are authorized to perform advanced interventions beyond the paramedic scope of practice, including advanced airway management, procedural sedation, administration of a broader range of pharmacologic agents, and complex clinical decision-making regarding on-scene management vs transport strategy. Paramedic-staffed ambulances provide the backbone of prehospital care and are staffed by highly trained EMS professionals capable of delivering Advanced Life Support, including defibrillation, basic airway techniques, and initial stabilization of critically ill or injured patients prior to hospital transport.

The ground EMS system is primarily composed of standard ambulance crews operating stretcher-capable emergency vehicles (approximately 3.5 tons), equipped for full prehospital care and patient transport. Both physician-staffed and paramedic-staffed crews use these type C emergency vehicles. In August 2024, as part of a pilot project, the rendezvous system was launched at three locations in the Slovak Republic.^2^ The rendezvous model is a two-tiered prehospital emergency care approach in which a physician-staffed unit (or in some countries “a physician-staffed rapid response vehicle”) is dispatched separately to meet with a standard paramedic ambulance crew at the scene. This system allows for targeted deployment of advanced medical expertise, improving physician resource allocation while maintaining rapid response capabilities.^3^

**Figure 1:**
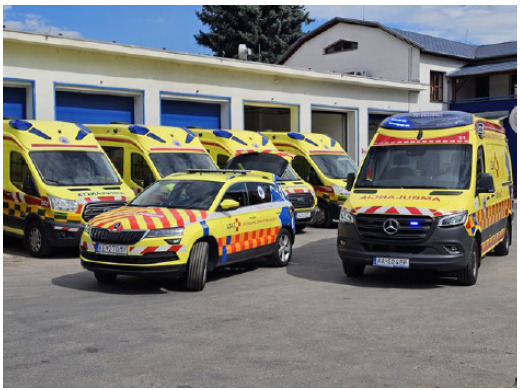
Comparison of vehicles used in a study of physician-staffed EMS in the Slovak Republic: on the left a smaller, faster “rendezvous” unit (Škoda Karoq), and on the right a standard emergency response vehicle (Mercedes Benz Sprinter).

Paramedic crews provide initial care in collaboration with the rendezvous unit, and hospital transport—if needed—can proceed with or without rendezvous involvement.^4^ Unlike standard physician- and paramedic-staffed crews, rendezvous units typically use passenger-type vehicles without transport capability, focusing instead on providing rapid, physician-level intervention (Figure 1). Although models with permanently assigned physician-staffed crews operating a large, transport-capable vehicle and the rendezvous system are well-established and frequently used in practice—either independently or concurrently—systematic scientific comparison in terms of effectiveness and impact on patient care remains limited in the literature. Our primary aim in this study was to analyze the impact of introducing a rendezvous crew into the prehospital EMS of the Košice region, Slovak Republic, on the time parameters of EMS responses. We hypothesized that the physician in a smaller, non-transport-capable vehicle that brings the physician-staffed crew to the prehospital scene would arrive faster than if the physician rode in the ambulance transport vehicle, and that the physician-staffed rendezvous crew would be available for the next deployment sooner.

**Population Health Research Capsule**

What do we already know about this issue?

*Physician-staffed EMS units may improve care for critical patients, but optimal deployment models to maximize response remain unclear*.

What was the research question?

*Does replacing a standard 3.5-ton emergency transport vehicle with a smaller rendezvous vehicle influence EMS response times and crew availability?*

What was the major finding of the study?

*Rendezvous implementation reduced response time by 0.77 minutes (95% CI, −1.01 to −0.53; P < .001) vs standard EMS transport*.

How does this improve population health?

*Faster physician response and shorter mission cycles may increase EMS availability, improving access to advanced care without additional staffing*.

## METHODS

### Setting

We conducted a retrospective, observational, cross-sectional study analyzing all primary responses by physician-staffed EMS crews in the Košice region, Slovak Republic, from January 2023–March 2025. The EMS Command and Control Centre of the Slovak Republic is a state-funded institution established by the Ministry of Health. It is organized into eight regional dispatch centres that operate under unified protocols and a shared information and communication technology infrastructure. Three stretcher-capable, physician-staffed ambulance units operate in the Košice region. While these units are primarily assigned to defined administrative districts, they may be dispatched outside their usual areas of service based on operational demand and closest unit availability, consistent with standard EMS dispatch practices. The rendezvous system was introduced in the study’s location in August 2024, when one standard physician-staffed transport-capable ambulance was replaced by a rendezvous unit.

### Objectives

The primary objective was to assess the impact of the rendezvous system on operational time parameters for physician-staffed EMS responses. Our primary outcome measure was response time to the patient’s location. Secondary outcomes included time spent on scene, time to transport initiation, and total time until physician-staffed crew availability. Appendix 1 presents technical specifications of the vehicles and stations used during the study period. Vehicles with transport capacity complied with the European technical standard EN 1789.^5^

### Inclusion Criteria

We included all primary emergency responses by the three physician-staffed EMS units in the Košice region (Sever, Západ, Staré Mesto) between January 2023–March 2025.

### Exclusion Criteria

We excluded responses outside the Košice districts and responses cancelled by the EMS Command and Control Centre (eg, 0- or 1-minute response intervals). We excluded the following time data: response times of 0 or 1 minute (due to cancellations or logging errors); response times > 60 minutes; or total response time duration > 180 minutes.

### Methodology

We extracted cases from the electronic database of the EMS Command and Control Centre, which is publicly accessible in accordance with the applicable legislation on access to information from public sources. Time parameters are recorded manually by crews and entered electronically using the “Fleet on Board” application (DATACAR, spol. s r.o., Slovak Republic). Upon receiving a dispatch call, the crew confirms the time of departure by pressing a button in the automatic vehicle location system. Arrival at the patient’s location is similarly confirmed. If transport is required or the crew becomes available for the next assignment, the appropriate status is selected in the same system. All timestamps are electronically logged and stored by the dispatch center.

To analyze and compare the impact of introducing the rendezvous crew into the EMS system, we divided the collected data into two periods: before and after the project’s implementation. For both periods, we performed an analysis of several operational time metrics. We defined response time to patient’s location as the time from dispatch order issued by the telecommunicator to the crew until arrival at the scene. The time spent with the patient on scene was measured from the crew’s arrival and the start of transport, or from the crew’s departure when transport was not necessary. We defined time to initiation of transport as the interval between crew arrival and start of transport (applicable only for cases requiring hospital transport). Finally, we measured the interval until the crew became available for the next dispatch—from dispatch order to the moment the crew was available for a new assignment—representing the entire operational cycle.

This study follows the “Strengthening the Reporting of Observational Studies in Epidemiology” guidelines and published recommendations for medical record review studies in emergency medicine.^6^ We applied explicit case selection criteria, with predefined inclusion and exclusion rules. All operational time intervals were clearly defined a priori. We obtained data from the national EMS Command and Control Centre electronic database, which uses a standardized application for timestamp entry by EMS crews. Because data abstraction was based on a uniform electronic system, no manual abstractors were employed and, thus, issues of abstractor training, performance monitoring, and interobserver reliability did not apply. The EMS crews entering timestamps were unaware of this study’s objectives. The sampling strategy involved all consecutive, physician-staffed responses during the defined study period, with exclusions for erroneous or incomplete records. Missing data were handled through complete case analysis, with predefined rules for exclusion of implausible values. We performed data analysis in May and June 2025. The Emergency Medical Services Command and Control Centre of the Slovak Republic (RZ 3090/2025) approved use of this data for our study.

### Data Analysis

We calculated time intervals using electronic timestamps recorded by EMS crews through the automatic vehicle location system. Only missions meeting the predefined inclusion criteria were included in the analysis. Categorical variables are reported as absolute counts and percentages, while time-based variables are presented in minutes. Continuous variables are reported as means with standard deviations or medians with interquartile ranges, as appropriate. The Shapiro-Wilk test indicated non-normal distributions for key variables. Differences in time parameters between the pre- and post-intervention periods (standard transport-capable ambulance vs fast-car rendezvous) were analyzed using the Mann-Whitney U test, with effect sizes expressed as rank-biserial correlation (r_e_). We used a Kruskal–Wallis test, as a non-parametric equivalent of one-way ANOVA, to compare physician-staffed EMS units across the six administrative districts of Košice, both before and after implementation of the rendezvous system.

Effect sizes for these comparisons are reported using epsilon squared (ɛ^2^). To adjust for potential confounding factors influencing response time, we calculated a multivariable ordinary least squares regression analysis. The outcome variable was the response time l from crew departure to arrival at the patient’s location, measured in minutes. The model included fixed effects for EMS crew, district, month, and dispatch priority, as well as a binary indicator (post-August 2024) capturing the effect of introducing the rendezvous model. Missing values were handled using a complete case analysis approach. No data imputation was performed, given the large volume of valid records, which ensured sufficient statistical power for most comparisons. A *P* value < .05 was considered statistically significant. We conducted analyses using JASP software v0.19.1 (University of Amsterdam, Netherlands), and ordinary least squares regression analysis in Python using the statsmodels package v0.14.2 (Python Software Foundation, Wilmington, DE).

## RESULTS

**Figure 2:**
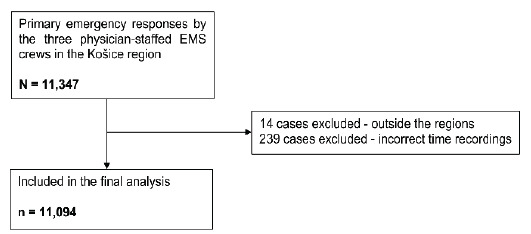
Flowchart of case selection and exclusions in a study of physician-staffed emergency medical services in Košice, Slovak Republic.

The inclusion criteria initially identified 11,347 cases for potential enrollment. Following review of the dataset, we excluded 253 cases: 14 due to responses outside the Košice region, and 239 due to erroneous time data samples. We included 11,094 cases in the final analysis (Figure 2).

Prior to implementation of the rendezvous model, physician-staffed transport units across all three districts demonstrated broadly comparable response intervals, on-scene times, and overall crew availability, with modest inter-district variation (Table 1). Following replacement of the physician-staffed standard transport unit with a rendezvous crew, response times across districts were slightly reduced, while on-scene and transport-initiation intervals increased modestly across all unit types. The most pronounced operational change was observed in availability of physician-staffed crews. After rendezvous implementation, the mean time-to- physician availability for the Sever district unit decreased substantially, whereas corresponding availability times increased in the Staré Mesto and Západ districts during the same period (Table 1).

To evaluate the impact of rendezvous implementation, we compared response times of standard transport units across six administrative districts of Košice, both before and after the system change. Before implementation, statistically significant differences in response intervals among the three standard transport units (Sever, Staré Mesto, and Západ) were found in five of the six districts (all *P* < .001), with effect sizes (ɛ^2^) ranging from small (0.032 in the Košice-okolie district) to moderate (0.117 in Košice 2). The only district without significant differences was Košice 3 (*P* = .81). After implementation of the smaller, faster rendezvous unit, differences in response intervals remained statistically significant in five of the six districts, with Košice 3 demonstrating the largest effect size (ɛ^2^ = 0.245). The only district without significant differences post-intervention was Košice-okolie (*P* = .65). The rendezvous unit consistently demonstrated shorter response intervals compared to the traditional transport units, particularly in Košice 1 and Košice 4 (Table 2).

Comparisons between the pre-intervention standard transport unit in Sever and the post-intervention rendezvous crew demonstrated consistent operational changes across most districts of Košice. Following implementation of the rendezvous model, response intervals were significantly shorter in all defined districts, with the largest relative improvements observed in Košice 2 and Košice 3 (absolute reductions of 2.64 and 2.15 minutes, and large effect sizes r_e_ = 0.616 and 0.419, respectively). No statistically significant change was detected in the undefined/border region (Table 3). The most substantial and uniform effect of RV deployment was observed in physician-staffed crew availability. Across all districts except the undefined/border region, the interval from mission start to availability for the next dispatch was significantly shorter for the RV Sever crew compared with the prior RLP model, with reductions exceeding 10 minutes in each district and the greatest absolute decreases occurring in Košice-okolie, Košice 3, and Košice 2 [reduction in Košice-okolie (−18.01 minutes, r_e_ = 0.444); followed closely by Košice 3 (−16.27 minutes, r_e_ = 0.458) and Košice 2 (−15.74 minutes, r_e_ = 0.442)]. In contrast, mean on-scene time was consistently longer for the RV Sever crew across all districts; however, these differences were modest in magnitude and did not reach statistical significance (ranging from +0.94 minutes in Košice 1 to +4.42 minutes in Košice 2, with intermediate increases of +3.72 minutes in Košice 3 and +1.65 minutes in Košice 4). Similarly, intervals from dispatch to transport initiation showed mixed, district-specific patterns, with small increases in some areas and modest reductions in others, none of which demonstrated a consistent or statistically significant trend. Detailed district-level measurements are provided in Table 3.

To adjust for potential confounding factors influencing response intervals, we conducted a multivariable ordinary least squares regression analysis. The outcome variable was the response interval from crew departure to arrival at the patient’s location, measured in minutes. The model included fixed effects for EMS crew, district, month, and dispatch priority, as well as a binary indicator (post-August 2024) capturing the effect of introducing the rendezvous model. The overall model fit was acceptable (adjusted *R*^2^ = 0.286), indicating that approximately 29% of the variance in response time was explained by the included predictors. Dispatch priority categories (compared to reference category “K” - critical; included “N” - urgent calls; and “M” - less urgent calls) were not significantly associated with differences in response time, suggesting that dispatch procedures in Košice did not systematically favor higher priority calls in terms of speed once other variables were accounted for. The implementation of the rendezvous system was associated with a statistically significant reduction in response time of −0.77 minutes (*P* < .001, 95% CI, −1.013 to −0.525), in comparison with all other relevant physician-staffed units, after adjusting for other covariates (Table 4).

## DISCUSSION

We evaluated the operational impact of implementing a physician-staffed, smaller, faster rendezvous vehicle on prehospital time parameters within the EMS system in Košice region, Slovak Republic. The rendezvous model was associated with a statistically significant reduction in response interval to patient location across most districts of Košice. While the overall regression-adjusted reduction in response time was −0.77 minutes (95% CI, −1.013 to −0.525), the absolute reductions observed at the district level ranged from 1.46–2.64 minutes. Post-intervention, modest reductions in response times were observed in nearly all physician-staffed crews, suggesting an effect that extended beyond the rendezvous Sever pilot unit. Implementation of the rendezvous unit likely improved response times of all standard transport units by redistributing workload, enhancing coverage flexibility, and enabling more targeted deployment of physician-level resources across overlapping districts; the exact mechanisms, however, require further study to be confirmed.

Although the observed reductions in response intervals were statistically significant, they should be interpreted primarily as indicators of operational efficiency rather than as direct evidence of improved patient outcomes. Evidence linking modest reductions in EMS response times to improved clinical outcomes is strongest for out-of-hospital cardiac arrest, whereas data supporting outcome benefits in other emergency conditions, including trauma, remain inconsistent or limited.^7^–^9^ The enhanced flexibility and better maneuverability capabilities of the lighter and smaller rendezvous vehicle may contribute to improved scene access, particularly in densely built or traffic-congested urban areas. The rendezvous unit was consistently faster in completing the entire operational cycle (from dispatch to availability for the next call), with reductions in total job time ranging from 11.12–18.01 minutes. This reflects greater system efficiency with a potential to increase EMS resource availability without additional staffing.

The largest reductions were seen in Košice-okolie and Košice 3, again pointing to the rendezvous model’s operational advantages in urban and peri-urban areas. The reduced interval until crew becomes available for the next dispatch is particularly relevant in resource-limited systems, where optimal crew utilization can directly impact population-level emergency coverage and physicians are more likely to be available for patients, who need their interventions the most. Modest reductions per mission in response and availability intervals (1–2 minutes) accumulate over hundreds of monthly missions, yielding several hours of additional operational capacity. This increased availability could help reduce queued calls during peak demand and improve overall system responsiveness.

Interestingly, the time interval spent with patients on scene was longer for the rendezvous crew compared to the standard transport crew in nearly all districts. The largest absolute differences were observed in Košice 2 (+4.42 minutes) and Košice 3 (+3.72 minutes), although neither reached statistical significance (*P* = .19 and *P* = .15, respectively). Across all districts, none of the observed differences reached statistical significance, despite meaningful numeric differences. Effect sizes were uniformly small, indicating only modest shifts in distribution. The trend toward longer scene intervals may reflect more comprehensive assessments or advanced interventions performed by the rendezvous unit physician, especially when a handover to the transporting paramedic crew is required. This aligns with the conceptual design of the rendezvous system, which prioritizes dispatch for the most severe cases, and a flexible, targeted deployment of physician expertise over transport speed.

A more detailed investigation and breakdown of the crew’s on-scene activities during patient care would be necessary to analyze the reasons for longer scene times. In contrast, interval to transport initiation did not demonstrate a consistent directional change between RLP and RV crews. Some districts (e., Košice 1) had longer initiation times for rendezvous crews, while others (eg, Košice 3, Košice-okolie) showed slightly faster performance. None of these differences reached statistical significance. The variability across districts may reflect local operational practices, differing scene complexities, or variable collaboration dynamics between physician and paramedic crews. The absence of a uniform pattern reinforces the idea that transport initiation time is multifactorial and likely influenced by factors beyond a crew type alone. The implementation of the rendezvous system was associated with a statistically significant reduction in response interval of −0.77 minutes (*P* < .001, 95% CI, −1.013 to −0.525), after adjusting for other covariates. This supports the hypothesis that the rendezvous model offers a more time-efficient deployment mechanism for physician-staffed units in the urban EMS context.

## LIMITATIONS

This study has several limitations that should be considered when interpreting the results. Its retrospective and observational nature precludes the establishment of causal relationships between the implementation of the rendezvous system and changes in EMS response intervals. One of the main challenges is the impracticality of randomizing emergency responses under real-world, prehospital-care conditions, which limits the ability to obtain robust causal evidence. While temporal comparisons were made before and after rendezvous initiation, there may be potential confounding factors influencing the data (eg, changes in staff composition, traffic patterns, or seasonal variation). Although comparisons were made between pre- and post-implementation periods, there may have been confounding variables influencing the results, such as changes in staff composition, traffic conditions, or seasonal variation. We attempted to mitigate their effect through the ordinary least squares regression analysis.

While efforts were made to ensure data quality, the study relied on a routinely collected operational dataset, which may be subject to inaccuracies in time logging. Despite excluding clearly erroneous data through defined criteria, some inaccurate entries may have remained in the final dataset. Although we examined multiple time parameters and intervals, we did not assess clinical outcomes, patient severity, or final diagnoses. Therefore, we could not determine whether observed changes in time metrics had any impact on patient care or outcomes. Additionally, the lack of imputation for missing data may have introduced bias if the data were not missing completely at random, although the large volume of complete cases likely reduced this risk. Lastly, the sample size for the rendezvous crew was considerably smaller than standard transport crews, particularly in certain districts, which may have limited the statistical power to detect small but clinically meaningful differences, even when absolute differences were notable.

### Generalizability

The findings of this study reflect EMS operations in the Košice region, Slovak Republic, which is characterized predominantly as an urban setting with a structured two-tier EMS system. This context may limit the generalizability of the results to other regions, especially those with different geographic, staffing, or operational and dispatch characteristics. However, the operational model examined—adding a physician-staffed rendezvous crew while maintaining existing physician-staffed standard transport units—is not unique to Košice. It may be relevant to other EMS systems exploring hybrid or tiered response strategies; however, extrapolation to systems with fundamentally different prehospital structures should be undertaken with caution, and further research in diverse settings is needed to strengthen the external validity of these findings.

## CONCLUSION

Our findings provide novel and relevant insights into the comparative performance of traditional physician-staffed crews with large, transport-capable ambulances, and a lighter, rendezvous model without transportation capacity, in the context of Slovak Republic. The rendezvous model was associated with shorter response intervals, improved crew availability, and enhanced operational efficiency. These findings highlight the potential of rendezvous systems to optimize physician deployment in urban EMS settings without requiring additional staffing. To our knowledge, this is the first data-driven evaluation of rendezvous implementation and contributes to the limited body of literature on hybrid EMS models. The results offer empirical support for expanding rendezvous deployment in comparable EMS environments as a strategy to improve operational efficiency. However, further research incorporating patient-centered clinical endpoints will be required to determine whether these operational gains translate into improved outcomes, and to better understand the cost-effectiveness and team-based implications of RV integration within broader EMS systems.

## Figures and Tables

**Table 1:** Comparison of different time parameters and intervals of physician-staffed ambulanced before and after introduction of the rendezvous system in a study of physician-staffed emergency medical services in the Košice region, Slovak Republic.

Before introduction of rendezvous system				After introduction of rendezvous system (mixed physician-staffed transport and rendezvous units)			
Response interval to patient’s location:							
	RLP Sever (N=2,807)	RLP Staré Mesto (N=2,759)	RLP Západ (N=2,823)		RV Sever (N=488)	RLP Staré Mesto (N=1,147)	RLP Západ (N=1,070)
Mean (min)	9.45	10.60	8.81	Mean (min)	9.22	10.03	8.43
SD (min)	5.42	6.14	5.82	SD (min)	5.54	5.67	34.63
Shapiro-Wilk	0.71	0.75	0.66	Shapiro-Wilk	0.85	0.74	0.70
*P* value of Shapiro-Wilk	< .001	< .001	< .001	*P* value of Shapiro-Wilk	< .001	< .001	< .001
Minimum	2.00	2.00	2.00	Minimum	2.00	2.00	2.00
Maximum	58.00	57.00	56.00	Maximum	49.00	52.00	51.00
Missing	0.00	12.00	16.00	Missing	7.00	2.00	3.00
Interval spent with the patient on scene:							
	RLP Sever (N=2,807)	RLP Staré Mesto (N=2,759)	RLP Západ (N=2,823)		RV Sever (N=488)	RLP Staré mMesto (N=1,147)	RLP Západ (N=1,069)
Mean (min)	31.03	26.48	28.00	Mean (min)	33.47	30.71	31.86
SD (min)	15.32	13.06	14.03	SD (min)	16.07	15.36	14.73
Shapiro-Wilk	0.90	0.93	0.91	Shapiro-Wilk	0.93	0.89	0.91
*P* value of Shapiro-Wilk	< .001	< .001	< .001	*P* value of Shapiro-Wilk	< .001	< .001	< .001
Minimum	0.00	0.00	0.00	Minimum	2.00	2.00	2.00
Maximum	149.00	133.00	163.00	Maximum	12600	152.00	160.00
Missing	0.00	18.00	12.00	Missing	0.00	0.00	1.00
Interval to transport initiation from the scene:							
	RLP Sever (N=1,807)	RLP Staré Mesto (N=1,714)	RLP Západ (N=1,907)		RV Sever (N=97)	RLP Staré Mesto (N=815)	RLP Západ (N=754)
Mean (min)	36.37	34.04	33.80	Mean (min)	37.90	37.46	35.98
SD (min)	11.42	10.98	11.36	SD (min)	13.20	12.11	10.63
Shapiro-Wilk	0.94	0.96	0.94	Shapiro-Wilk	0.95	0.94	0.96
*P* value of Shapiro-Wilk	< .001	< .001	< .001	*P* value of Shapiro-Wilk	0.001	< .001	< .001
Minimum	10.00	7.00	6.00	Minimum	12.00	9.00	7.00
Maximum	130.00	100.00	161.00	Maximum	84.00	111.00	94.00
Missing	0.00	0.00	0.00	Missing	0.00	0.00	0.00
Interval until crew is available for the next dispatch:							
	RLP Sever (N=2,807)	RLP Staré Mesto (N=2,759)	RLP Západ (N=2,823)		RV Sever (N=488)	RLP Staré Mesto (N=1,146)	RLP Západ (N=1,070)
Mean (min)	60.78	57.36	57.91	Mean (min)	49.58	64.25	62.67
SD (min)	22.08	22.94	22.15	SD (min)	23.03	24.08	19.70
Shapiro-Wilk	0.96	0.94	0.96	Shapiro-Wilk	0.91	0.94	0.96
*P* value of Shapiro-Wilk	< .001	< .001	< .001	*P* value of Shapiro-Wilk	< .001	< .001	< .001
Minimum	9.00	9.00	4.00	Minimum	7.00	9.00	10.00
Maximum	178.00	179.00	163.00	Maximum	19000	211.00	183.00
Missing	3.00	2.00	2.00	Missing	0.00	1.00	0.00

*RLP*, standard transport unit; *RV*, passenger car rendezvous unit.

**Table 2:** Response time of physician-staffed crews to the individual districts of Košice in a study of physician-staffed emergency medical services in the Košice region, Slovak Republic.

Districts	Before introduction of rendezvous system (standard transport units only)											

	RLP Sever (N = 2,807)			RLP Staré Mesto (N = 2,759)			RLP Západ (N = 2,823)			P	ɛ ^2^	95% CI
	N	Mean time	SD	N	Mean time	SD	N	Mean time	SD			
Košice 1	1,132	9.3	4.5	449	10.5	5.2	667	8.9	4.9	< .001	0.039	0.024 – 0.056
Košice 2	307	9.2	5.1	356	8.8	5.2	1472	7.4	5.1	< .001	0.117	0.094 – 0.145
Košice 3	301	10.6	4.1	324	10.6	3.0	89	10.8	4.4	.81	0.001	0.001 – 0.012
Košice 4	739	6.8	4.0	1,021	8.2	5.1	233	8.2	5.3	< .001	0.048	0.031 – 0.072
Košice-surrounding districts	215	18.4	6.5	526	16.4	6.3	247	17.2	6.2	< .001	0.032	0.016 – 0.054
Undefined/border region	113	8.3	4.5	83	11.3	8.8	115	7.8	4.7	< .001	0.058	0.019 – 0.121

Districts	After introduction of rendezvous system (mixed standard transport and smaller faster rendezvous units)											

	RV Sever (N = 488)			RLP Staré Mesto (N = 1,147)			RLP Západ (N = 1,070)			P	ɛ ^2^	95% CI
	N	Mean time	SD	N	Mean time	SD	N	Mean time	SD			

Košice 1	116	7.3	2.6	225	10.1	4.2	337	8.5	3.2	< .001	0.104	0.067 – 0.160
Košice 2	73	7.1	2.2	170	8.1	2.6	487	7.4	4.5	< .001	0.051	0.026 – 0.086
Košice 3	52	7.9	1.9	148	11.2	5.1	55	11.6	6.3	< .001	0.245	0.180 – 0.336
Košice 4	110	5.4	2.6	404	7.4	4.3	92	7.1	2.3	< .001	0.095	0.047 – 0.154
Košice-surrounding districts	127	16.2	5.7	170	17.0	6.8	65	15.9	5.6	.65	0.002	0.001 – 0.032
Undefined/border region	10	6.6	2.3	30	10.1	5.5	34	6.4	2.4	< .001	0.182	0.077 – 0.386

*RLP*, standard transport unit; *RV*, passenger car rendezvous unit; *SD*, standard deviation.

**Table 3:** Comparison of operational intervals for standard transport vs. rendezvous units, stratified by districts of Košice in a study of physician-staffed emergency medical services in Slovak republic.

Districts	Comparison between standard transport and rendezvous unit Response intervals stratified by districts of Košice							

	N (RLP)	N (RV)	U	RLP (mean)	RV (mean)	Absolute difference	*P*	Effect size (re)
Košice 1	1.132	116	89,628.50	9.32	7.34	−1.99	< .001	0.365
Košice 2	307	73	15,899.00	9.21	7.06	−2.15	< .001	0.419
Košice 3	301	52	12,643.50	10.56	7.92	−2.64	< .001	0.616
Košice 4	739	110	54,509.00	6.82	5.36	−1.46	< .001	0.341
Košice-surrounding districts	215	127	16,805.00	18.40	16.21	−2.19	< .001	0.231
Undefined/border region	113	10	738.50	8.27	6.60	−1.67	.11	0.307
Total	2,807	488						

Districts	Comparison between standard transport and rendezvous Sever units Interval until crew is available for the next dispatch stratified by districts of Košice							

	N (RLP)	N (RV)	U	RLP (mean)	RV (mean)	Absolute difference	*P*	Effect size (re)

Košice 1	1,132	116	95,286.50	60.53	46.02	−14.52	< .001	0.439
Košice 2	307	73	16,493.00	62.48	46.73	−15.74	< .001	0.442
Košice 3	301	52	11,375.50	63.58	47.31	−16.27	< .001	0.458
Košice 4	739	110	55,490.00	56.27	45.14	−11.12	< .001	0.353
Košice-surrounding districts	215	127	20,185.50	74.14	56.14	−18.01	< .001	0.444
Undefined/border region	113	10	763.00	55.67	45.50	−10.17	.07	0.350
Total	2,807	488						

Districts	Comparison between standard transport and rendezvous Sever units Interval spent with the patient on scene stratified by districts of Košice							

	N (RLP)	N (RV)	U	RLP (mean)	RV (mean)	Absolute difference	*P*	Effect size (re)

Košice 1	1,132	116	61,937.00	30.76	31.70	0.94	.31	0.056
Košice 2	307	73	10,099.50	31.10	35.52	4.42	.19	0.075
Košice 3	301	52	6,853.50	31.36	35.08	3.72	.15	0.087
Košice 4	739	110	38,718.50	32.18	33.83	1.65	.42	0.059
Košice-surrounding districts	215	127	12,385.50	31.33	33.16	1.83	.15	0.065
Undefined/border region	113	10	502.50	25.39	27.20	1.81	.57	0.189
Total	2,807	488						

Districts	Comparison between standard transport and rendezvous Sever units Interval to transport initiation stratified by districts of Košice							

	N (RLP)	N (RV)	U	RLP (mean)	RV (mean)	Absolute difference	*P*	Effect size (re)

Košice 1	723	24	7,331.50	35.80	39.79	3.99	.12	0.119
Košice 2	213	13	1,518.0	36.05	34.54	−1.51	.56	0.164
Košice 3	199	9	981.50	38.89	37.33	−1.55	.63	0.195
Košice 4	468	21	5,454.50	35.09	34.05	−1.04	.39	0.128
Košice-surrounding districts	127	26	1,751.00	43.07	40.52	−2.55	.42	0.126
Undefined/border region	77	4	164.00	32.77	31.00	−1.77	.84	0.289
TOTAL	1,807	97						

*RLP*, standard transport unit; *RV*, passenger car rendezvous unit; *re*, random effects.

**Table 4:** Multivariable ordinary least squares regression analysis of factors associated with response times in a study of physician-staffed emergency medical services in the Košice region, Slovak Republic.

Variable	Coef.	Std. Err.	t	*P* value	95% CI
Intercept	9.3295	0.199	49.6	<.001	[8.961, 9.698]
Priority (ref: K)					
N	−0.1915	0.258	−0.74	.458	[−0.697, 0.314]
M	0.1552	0.118	1.32	.187	[0.075, 0.386]
Crew (ref: RLP Server)					
RLP Staré Mesto	0.7202	0.117	6.15	<.001	[0.491, 0.950]
RLP Západ	−0.1925	0.124	−1.56	.120	[−0.435, 0.050]
District (Ref: Košice 1)					
Košice 2	−1.3560	0.130	−10.47	<.001	[−1.610,−1.102]
Košice 3	1.1479	0.176	6.52	<.001	[0.803,1.493]
Košice 4	−2.0048	0.131	−15.33	<.001	[−2.261,−1.749]
Košice-okolie	7.4044	0.158	46.74	<.001	[7.094, 7.715]
Month Fixed Effects	Included (not displayed)				
Post-Intervention (Aug 2024+)					
RV implemented	−0.7692	0.124	−6.18	<.001	[−1.1013, −0.525]

Model summary: N = 10,687; R^2^ = 0.287; Adjusted R^2^ = 0.286
